# Who uses technical aids in old age? Exploring the implementation of technology-based home modifications in Europe

**DOI:** 10.3389/fpubh.2023.1130177

**Published:** 2023-03-30

**Authors:** Laura I. Schmidt, Melanie Wagner, Hanna A. Büßecker, Annette A. Franke

**Affiliations:** ^1^Institute of Psychology, Heidelberg University, Heidelberg, Germany; ^2^Max Planck Institute for Social Law and Social Policy, Max Planck Society Munich, Munich, Germany; ^3^Department of Social Work, Protestant University of Applied Sciences Ludwigsburg, Ludwigsburg, Germany

**Keywords:** aging in place, home modifications, technical aids, functional abilities, social network, mental health, internet use among older adults, mobility limitations

## Abstract

**Introduction:**

Home modifications and features, e.g., handrails or ramps for people using wheelchairs, should allow residents with functional limitations to maintain social participation, health, and wellbeing for aging in place. However, there is little evidence in relation to the individual characteristics shaping this implementation of technology-based home modifications. Current studies often focus on describing the distribution of certain implementations in households but do not provide information on factors predicting the implementation or detailed and multifaceted data on associations with characteristics of the older user. This article, therefore, examines the use of well-established technological aids and home modifications (e.g., ramps, handrails, automatic doors, bathroom or kitchen modifications, chair lifts, and alerting devices) in the households of older adults in Europe. We refer to Lawton's and Nahemow's concept of personal-environment fit and describe the use of technical aids across 18 countries, analyze associations with individual characteristics and social resources, and compare those associations and variance explanation between older adults in their third age (“young-old”, 65–79 years) and older adults in their fourth age (“old-old”, 80+).

**Methods:**

Drawing on representative data from the Survey of Health, Ageing, and Retirement in Europe (SHARE), wave 6, a total of N = 38,553 older adults aged 65–105 years (M = 74.4 years, SD = 7.1; 55% women) were analyzed by performing hierarchical logistic regression analyses.

**Results:**

Indicators of functioning explained the highest proportion of variance, followed by social resources, and variance explanation was higher for the fourth age than for the third age. In particular, older adults with physical limitations, a larger social network, and those who received care from a child outside the household were more likely to have home modifications installed.

**Discussion:**

The study provides an overview of associations of diverse variables with assistive devices and modifications in the home and can serve as a starting point for public health activities concerning the heterogeneity of people aged 65 years and older.

## 1. Introduction

“Aging in place” and “aging in the community”—the ability to live safely, independently, and comfortably in one's own home and community, regardless of age, income, or competence level—have gained public interest and become key topics for older individuals as well as in healthcare policies (1, 2). The creation of age-friendly environments entails several dimensions such as transport infrastructure, safety in the community, accessibility to houses and public spaces, and universal design (3, 4). In this context, home modifications and features, e.g., handrails or ramps for people using wheelchairs, should allow residents with functional limitations to maintain activities in their daily life. The WHO (5) also underlines the possibilities of access to the internet and assistive technologies to maintain social participation, information, and quality of life for older people in their homes. Expectations are high that technology has the potential to facilitate health prevention, independence at home, and overcoming challenges in healthcare, especially as we have now entered the so-called Decade of Healthy Aging (2021–2030) (6). According to statistics from the European Commission (7), during the next 50 years, the ratio of Europeans aged 65 years and older will increase from 20% today to 30% in 2070. In addition, 49.7% of the EU population aged 65 years and older report moderate or severe difficulties with at least one everyday activity (8).

Indeed, a growing number of research demonstrates the use and potential of technology-based home modifications, although studies also indicate that older people may face barriers when implementing these technologies. Previous studies found that the modification of grab bars is used in particular, followed by shower seats, raised toilet seats, and grab bars near the toilet for persons aged 52 or older (9) or aged 65 or older (10). These features can be classified as technical aids with a low level of digitalization. With regard to the impact of technical aids (e.g., ramps and alarm buttons) on functional health, recent studies provide evidence that modifications in the home reduce difficulties in performing (instrumental) activities of daily living. Liu and Lapane (11) examined data from two waves (*N* = 9,447) of the Second Longitudinal Study of Aging, a representative study of older noninstitutionalized adults aged 70 years and older from the United States. The results show that people aged 70 years and older who had modifications in their home at the baseline measurement were less likely to experience a worsening of their functional abilities after 2 years compared with older adults without modifications. Using data from the Survey of Health, Ageing, and Retirement in Europe (SHARE) for adults aged 60 years and older, Wu et al. (12) analyzed the extent to which home modifications (e.g., wide doors and grab bars) were related to health status after 3 years of implementation. Results showed that adding one modification to the house was associated with a reduction in the risk of falling by about 1.3%. In addition, the Swedish study from Petersson et al. (13) explored improvements due to the implementation of technical aids in the household. Participants who reported difficulties and feelings of insecurity in performing various (instrumental) activities of daily living (e.g., climbing stairs) at the first measure point reported better performances 2 months after the intervention.

However, there is little evidence in relation to the individual characteristics shaping this implementation of technology-based home modifications. Current studies often focus on describing the distribution of certain implementations in households but do not provide information on factors predicting the implementation. Regarding the type of technology, information and communication technologies (ICT) are often the focus of attention. Having a deeper understanding of the characteristics which influence the probability of home modifications can be useful to address more effective and sustainable interventions for aging in place and in the community.

### 1.1. Technical aids in the household—Characteristics and resources of older users

First, studies with European data found that a significant number of older adults make modifications to their households with country-specific differences. Wu et al. (12) reported that 22% of Europeans modified their households and that the use of assistive devices was higher in the countries studied from Western Europe (e.g., France and Germany) than in Southern European countries (e.g., Spain and Italy) (25 and 10%, respectively). A longitudinal study (14) examined older adults from five European countries (Sweden, Germany, United Kingdom, Latvia, and Hungary) regarding the use of assistive devices. They analyzed data from the European research project Enabling Autonomy, Participation, and Wellbeing in Old Age: The Home Environment as a Determinant for Healthy Aging (ENABLE AGE). Here, too, it was found that many older people had a desire for supportive modifications but did not act on them. Approximately 24% of Europeans involved in the study (*N* = 1,918) reported an unmet need for adaptations (e.g., aids for showering) (14).

Building on *socio-demographic characteristics*, some studies suggest that women were more likely to have home modifications than men (10, 15, 16). In addition, the probability of modifications increases with age to successfully adapt to limitations in mobility (17, 18). The European study from Wu et al. (12) confirmed that older adults aged 80 years and older (33%) were more likely to use assistive devices compared to adults between 60 and 70 years of age (22%). With regard to the influence of educational background and income, studies provide largely varying results. While some studies underline a higher educational level as a significant predictor of a higher number of applied aids (10, 19, 20), other research indicates a higher probability of modifications for persons with lower education especially regarding features to maintain mobility (21). In the study of Ishigami et al. (21) also a lower income was associated with higher use of assistive devices. However, considering the interaction of income and health status, it can be assumed that persons with higher income had fewer functional limitations, and accordingly, their need for assistive devices was lower (15).

*Social support* is considered an important resource for aging in place as well as aging in the community (22). Receiving help from people in their social network also indicates to enable the implementation and use of technical aids (10, 23, 24) Partners, children, or peers may recommend or discourage the use of certain devices based on their preferences and opinions. Thus, technical features such as alarm sensors may be used to reassure relatives not living close by (24). In particular, informal caregivers' need for information (e.g., seeking information to ensure the safety of the care recipient at home) and their perception of fall risk (e.g., due to past falls) correlates positively with household modifications (17, 25, 26).

Only few studies explored the associations between *mental health* and the use of assistive devices among older persons in their homes and results appear to be inconsistent. While some studies found no significant relation between mental health problems (e.g., depression, guilt, and sleep problems) and the use of assistive devices (12, 27), the results of other studies show that, for example, feelings of loneliness and use of mobility aids (walking sticks) were positively correlated (21). On the other hand, according to a US study, depressive symptoms of people aged 65 years and older decreased the likelihood of mobility aids (i.e., walkers) by about 25% while at the same time, the likelihood of personal assistance was increased (28). Moreover, a cross-sectional Swedish study found that the number of physical environmental barriers as well as lack of accessibility (e.g., dependence on mobility aids, functional limitations, narrow doors, and lack of grab bars) were negatively related to life satisfaction (29).

*Functional health*, along with sociodemographic variables, is the most commonly studied predictor of older adults' installation of home modifications. Previous research classifies a limited level of physical functioning as a facilitating factor for use of technological modifications in the home. Several studies show that older adults with multiple chronic conditions are more likely to use assistive devices (10, 21, 30).

In addition, a variety of studies provide evidence that limitations in (instrumental) activities of daily living (IADL/ADL) are related to a higher likelihood of the implementation of assistive technology devices in the homes of older adults (10, 12, 20, 27, 31, 32). For example, results of Pressler and Ferraro's study show that lower body impairments, in particular, that lead to severe difficulties in activities of daily living, such as climbing stairs, significantly predicted the number of assistive devices used (20).

In addition, recent studies indicate that subjectively assessed poor health is also associated with the implementation of assistive technology. Data from a Canadian longitudinal study (21) found that older adults aged 65 years and older with poor perceived health status increased their use of assistive technology. According to this study, 60% of women and 50% of men used an assistive device to support mobility, such as walkers. In contrast, 9% of women and 8% of men with excellent perceived health status used assistive devices.

Thus far, there is a significant lack of research examining the extent to which the *use of information and communication technologies* such as the internet can also be transferred to the use of assistive devices in the home (e.g., alarm buttons). However, it could be inferred that global confidence in one's own ICT skills can also apply to household modifications.

### 1.2. The interconnection of person, environment, and household modifications

Theoretically, the environmental press theory by Lawton and Nahemov (33) is suited to explain the connections between a person‘s exposure to technology and contextual conditions. The theory highlights the fit between the individual competences (e.g., physical health and cognitive competences) and the environment (e.g., home, social interactions, and neighborhood) and their interaction with each other. The level of the person includes the ability and willingness, while the environment—as a special but also socially created construct—gives the framework of what is possible or should be done. A successful balance between person and environment means that there are no significant disparities between both levels of individual competences and environmental demands. Thus, persons are influenced in their being and thinking by their environment but are also agents for changing the environment to overcome disparities. In particular, the theory conceptualizes the influence of the environment on “wellbeing” in old age or on “aging well”. The authors argue that the behavior of older people is increasingly determined or shaped by environmental conditions with age, as declining resources (physical, cognitive, or social) make it more difficult to change those conditions or overcome certain barriers (33). Thus, environmental conditions gain importance for aging independently and aging in place. “Aging well” means an adequate fit of resources and environment in an interplay of its physical, social, and technical characteristics and individual needs. According to this approach, the situation can become imbalanced by an increasing need for care, when own resources and activities of daily living contrast with the circumstances in the environment, and individuals are not able to compensate or modify the situation.

Hence, the environment may influence the vulnerability of personal wellbeing and health if, for example, there are physical barriers or infrastructural deficits. However, it can also strengthen health and wellbeing in old age if, for example, technical or social support can compensate for age-related impairments (34, 35). The use of assistive devices and implementation of technical aids implies an active adaptation process in order to maintain one's own independence and continue to feel “at home” in a balance of agency (level of action and modification) and belonging (meaning, identity, and familiarity) (35).

### 1.3. Research aims

The present study examined the use of well-established assistive devices and home modifications (e.g., ramps, handrails, automatic doors, bathroom or kitchen modifications, chair lifts, and alerting devices) in the households of older adults in Europe by drawing on representative data from the Survey of Health, Ageing, and Retirement in Europe (SHARE), wave 6 (36). Referring to Lawton‘s and Nahemow‘s (33) concept of person-environment fit, we explored the contribution of sociodemographic characteristics, social resources, mental health, health and functional abilities, and internet usage and computer competence in explaining if home modifications are installed in the household. Moreover, we aimed to compare those associations and variance explanations between older adults in their third age (“young-old”, 65–79 years) and older adults in their fourth age (“old-old”, 80+) (37, 38). We are using this distinction between both age groups given that frailty and functional limitations are statistically more associated with people aged 80 years and older. At the same time, we are aware of the heterogeneity and blurred boundaries in the transition from young-old to old-old.

## 2. Method

### 2.1. Data and participants

Data for the present study derive from the sixth wave of SHARE (Survey of Health, Ageing, and Retirement in Europe), a longitudinal panel survey including participants from the age of 50 years and older plus their co-residing partners, independent of age (36, 39–41). The fieldwork of the sixth wave of SHARE was completed in November 2015. Background information gathered in former SHARE waves was added by using the *easy* SHARE dataset (36, 42). Interviews include questions regarding health, functional abilities, household composition, economic situation, work, volunteering, and social or psychological variables. They are conducted biannually in a variety of European countries. All participants provided informed consent at the beginning of the computer-assisted personal interview (CAPI).

We restricted our analyses to those aged 65+ years. In total, data of *N* = 38,553 individuals from 18 countries (Austria, Belgium, Croatia, Czech Republic, Denmark, Estonia, France, Germany, Greece, Israel, Italy, Luxemburg, Poland, Portugal, Slovenia, Spain, Sweden, and Switzerland) were analyzed. Participants‘ age ranged from 65 to 105 years (M = 74.4 years, SD = 7.1) and 55% were women. All participants were community-dwelling older adults and did not live in nursing homes or comparable institutions.

### 2.2. Study variables

Our target variable implementation of technology-based home modifications was based on the question, which special features were present in the homes of participants (i.e., ramps or street level entrances, handrails, automatic or easy open doors or gates, bathroom or toilet modifications, kitchen modifications, chair lifts or stair glides, alerting devices such as button alarms or detectors, and other modifications). Due to the extremely skewed distribution (also after log transformation), we decided to use a dichotomized outcome variable with the values 0 = no modifications/special features and 1 = modifications/special features implemented. We included five blocks of predictor variables (1. background information, 2. social resources, 3. mental health, 4. health and functional abilities, and 5. internet usage and computer competence) to estimate the probability of having home modifications installed.

#### 2.2.1. Background information

As background information, we included *gender* (0 = male, 1 = female), *age, education*, and *household income* (in €). For education, the country-specific categories were classified according to the International Standard Classification of Education (ISCED-97) and recoded into low/medium (ISCED 1 to 4) and high (ISCED 5 and 6).

#### 2.2.2. Social resources

Social resources were captured via four constructs, namely *single household* (no = 0, yes = 1), *number of children* (biological and non-biological), *social network size* (0–7), and *receiving care from a child outside the household* (no = 0, yes = 1). Social network size was assessed by asking respondents to name up to seven people with whom they discuss important things (43).

#### 2.2.3. Mental health

To indicate the mental health of participants, we included *quality of life*, assessed via the CASP-12 scale (44) which includes the domains of control, autonomy, self-realization, and pleasure (12 items, range 12 to 48, higher scores indicating higher quality of life). Moreover, *depressive symptoms* were assessed with the Euro-D Scale (45) (16 items, range 0–12 = very depressed), and *loneliness* using the Three-Item Loneliness Scale (46) (three items, range 3–9, higher scores indicating higher loneliness).

#### 2.2.4. Health and functional abilities

Seven indicators were used to address health and functional abilities. We included the *body mass index (BMI), self-rated health*, a single item, ranging from poor (=1) to excellent (=5), *number of diseases* (e.g., hypertension, cancer, diabetes mellitus, range: 0 to 9), and *maximal grip strength* (range 1–98 kg), (47). To describe the number of limitations with *activities of daily living*, the ADL index was included (48). The modified version used in SHARE includes six activities (49). Thus, scores range from 0 to 6 with higher scores indicating more difficulties with these activities. To indicate *mobility limitations*, respondents could name up to 10 limitations in everyday activities related to mobility, e.g., walking for 100 m or picking up a small coin from a table. A dichotomous variable was used, differentiating people with no limitations (=0) from those with any limitation (=1). Moreover, *fear of falling* (no = 0, yes = 1) was included.

#### 2.2.5. Internet usage and computer competence

The last block of predictors included *internet usage*, namely, if participants had used the internet at least once during the last 7 days (no = 0, yes = 1), and *computer skills* on a 5-point Likert scale which was recoded for our analyses with higher scores indicating higher self-rates competence (1 = poor; 5 = excellent).

Descriptive statistics for predictor variables including means, standard deviations, and range of study variables are provided in Table 1.

**Table 1:** Descriptive statistics for the total sample (65+ years).

**Variable**	***M* or *%***	***SD***	**Range**
**Background information**			
Gender (% female)	55.0%		
Age	74.4	7.1	65–105
Education^a^	18.9%		
Household net income (€)	23,828.3	30,049.9	0–2.526,411.0
**Social resources**			
Single household (% yes)	28.0%		
Number of children	2.2	1.4	0–19
Social network size	2.6	1.6	0–7
Care from child outside household (% yes)	24.0%		
*Mental Health*			
Quality of Life^b^	36.5	6.5	12–48
Depressive symptoms^c^	2.6	2.3	0–12
Loneliness^d^	4.1	1.5	3–9
**Health and functional abilities**			
Body mass index	27.0	4.5	12–99
Self-rated health^e^	2.6	1.0	1–5
Number of chronic diseases	1.5	1.3	0–9
Maximal grip strength	30.4	10.9	1–98
Activities of daily living (ADL)^f^	0.36	0.9	0–5
Mobility limitations (% yes)	60.2%		
Fear of falling (% yes)	18.3%		
**IT usage and competence**			
Internet usage (% yes)	33.8%		
Computer skills^g^	2.2	1.3	1–6

N = 38,553.

a Proportion with higher education (ISCED 5, 6);

b CASP-12 scale, higher scores indicate higher quality of life;

c EURO-D scale, higher scores indicate higher depression;

d Three-Item Loneliness Scale, higher scores indicate higher loneliness;

e higher scores indicate better health;

f ADL index, higher scores indicate more difficulties with ADL;

g higher scores indicate better self-rated computer skills.

### 2.3. Statistical analyses

Binomial logistic regression analyses were performed to determine the effects of background variables, social resources, mental health, health and functional abilities, and computer competencies to predict the likelihood of having technical aids and modifications in the household. Statistical analyses were performed using SPSS version 27.0.

Key assumptions regarding binomial logistic regression analyses were met (i.e., linear relationship using the Box-Tidwell procedure, no outliers, no multicollinearity or auto-correlation) for the total sample (65+ years) as well for the analyses of the two subsamples in the third age (65–79 years) and fourth age (80+ years).

## 3. Results

In the total sample (65+ years), 13% of households had at least one technical modification/special feature installed. Bathroom or toilet modifications (6%) and handrails (5%) were the most common modifications. With regard to age groups, the percentage of having at least one modification at home was lower in the third age (65–79 years) with 11% compared to the fourth age (80+) with 18%.

Logistic regression analyses revealed a variance explanation of approximately 9.2% (Nagelkerke‘s R^2^) in the total sample, with a higher variance explanation (13.3%) for the fourth-age subsample and only 7.1% among the third-age participants. As expected, the block containing health variables and functional indicators revealed the highest contribution in the total sample and both age groups, followed by social resources.

Odds ratios and confidence intervals for every single predictor are depicted in Table 2 for the total sample. For example, with respect to sociodemographic variables, older age and a higher household net income increased the likelihood of home modifications, whereas gender was not a relevant predictor. For the second block which contained social resources, the most important predictors were social network size, with each additional person named as a close confidant increasing the likelihood of implementation by 27% and receiving care from a child outside the household, which implied a 60% higher chance for home modifications. Associations for the third block of mental health resources were lower, with loneliness showing no significant effect and rather smaller effects on quality of life and depression. Within the fourth block which comprised health and functional indicators, higher limitations in activities of daily living, having mobility limitations, and reporting fear of falling most prominently increased the likelihood of having (technical) features installed with increased likelihood between 33% and 44%. Significant but small effects were found for BMI, health, and grip strength. Finally, internet usage and higher self-reported computer skills did increase the odds of home modifications, although the contribution to the variance explanation of this last block was not substantial (0.5%).

**Table 2:** Logistic regression analysis explaining the implementation of technology-based home modifications for the total sample (65+ years).

**Predictor**	**OR**	**95% CI**
**Background information**		
Gender^a^	0.91	0.81–1.03
Age	**1.24**	1.19–1.31
Education^b^	**0.90**	0.81–0.99
Household net income	**1.14**	1.10–1.19
**Social resources**		
Single household^c^	0.99	0.91–1.09
Number of children	**1.05**	1.01–1.10
Social network size	**1.27**	1.23–1.32
Care from child outside household^c^	**1.60**	1.45–1.76
**Mental health**		
Quality of life^d^	**1.18**	1.12–1.25
Depressive symptoms^e^	**1.13**	1.07–1.19
Loneliness^f^	0.98	0.94–1.03
**Health and functional abilities**		
Body mass index	**1.02**	1.01–1.03
Self-rated health^g^	**1.06**	1.01–1.12
Number of chronic diseases	1.03	0.99–1.07
Maximal grip strength	**0.93**	0.88–0.99
Activities of daily living (ADL)^h^	**1.33**	1.26–1.40
Mobility limitations^i^	**1.44**	1.31–1.59
Fear of falling^c^	**1.44**	1.30–1.59
**IT usage and competence**		
Internet usage^j^	**1.21**	1.07–1.37
Computer skills^k^	**1.11**	1.04–1.18
**Model Fit**	χ^2^ = **1094.484 (20)**, ***p***<**0.001**,	
	**Nagelkerke‘s** ***R**^2^*= **0.092**	

N = 18,892; Method = Enter. OR, Odds Ratio; CI, Confidence interval.

Significant Odds Ratios in bold (p < 0.05).

a 0 = male, 1 = female;

b = low/medium (ISCED 1–4, 95, 97), 1 = high (ISCED 5, 6);

c 0 = no, 1 = yes.

d CASP-12 scale, higher scores indicate higher quality of life;

e EURO-D scale, higher scores indicate higher depression;

f Three-Item Loneliness Scale, higher scores indicate higher loneliness;

g higher scores indicate better health;

h ADL index; higher scores indicate more difficulties with ADL;

i Limitations in mobility, arm function, and fine motor skills, 0 = no, 1 = yes;

j Internet use within the last 7 days, 0 = no, 1 = yes;

k higher scores indicate better self-rated computer skills.

Comparing the two subsamples, some variables were of stronger importance in the fourth age (see Table 3), with larger effects for higher fear of falling (37% higher likelihood in the third age vs. 59% in the fourth age), having mobility limitations (39% in third age vs. 66% in fourth age), or lower grip strength, which was not significant in the younger sample (4% higher likelihood) but increased the likelihood of having home modifications in the older sample (15% higher likelihood). On the other hand, internet usage was only of importance among the young-old but was insignificant among the old-old.

**Table 3:** Logistic regression analyses explaining the implementation of technology-based home modifications by age group.

	**65–79 years (*****N*** = **14,852)**		**80**+ **years (*****N*** = **4,040)**	
**Predictor**	**OR**	**95% CI**	**OR**	**95% CI**
**Background information**				
Gender^a^	0.92	0.80–1.06	0.89	0.71–1.11
Age	**1.21**	1.12–1.31	**1.35**	**1.15–1.58**
Education^b^	**0.88**	0.78–0.99	0.93	0.75–1.17
Household net income	**1.14**	1.10–1.19	**1.16**	1.04–1.28
**Social resources**				
Single household^c^	0.97	0.87–1.08	1.01	0.86–1.20
Number of children	1.03	0.98–1.08	**1.10**	1.01–1.19
Social network size	**1.26**	1.20–1.31	**1.34**	1.23–1.45
Care from child outside household^c^	**1.65**	1.47–1.86	**1.49**	1.26–1.76
**Mental health**				
Quality of life^d^	**1.14**	1.07–1.22	**1.29**	1.16–1.44
Depressive symptoms^e^	**1.13**	1.07–1.20	**1.12**	1.01–1.24
Loneliness^f^	0.99	0.94–1.05	0.97	0.89–1.06
**Health and functional abilities**				
Body mass index	**1.01**	1.01–1.02	**1.04**	1.02–1.06
Self-rated health^g^	1.04	0.98–1.11	**1.13**	1.02–1.25
Number of chronic diseases	1.04	0.99–1.09	1.01	0.93–1.09
Maximal grip strengh	0.96	0.90–1.04	**0.85**	0.74–0.97
Activities of daily living (ADL)^h^	**1.31**	1.23–1.41	**1.35**	1.24–1.47
Mobility limitations^i^	**1.39**	1.24–1.54	**1.66**	1.33–2.08
Fear of falling^c^	**1.37**	1.20–1.55	**1.59**	1.34–1.89
**IT usage and competence**				
Internet usage^j^	**1.26**	1.10–1.45	1.01	0.75–1.38
Computer skills^k^	**1.10**	1.03–1.18	**1.17**	1.01–1.36
Model Fit	χ^2^ = 638.40 (20), *p* < .001		χ^2^ = 376.55 (20), *p* < .001	
	Nagelkerke‘s *R^2^*= .071		Nagelkerke's *R^2^*= .133	

Method = Enter; OR, Odds Ratio; CI, Confidence interval.

Significant Odds Ratios in bold (p < 0.05).

a 0 = male, 1 = female;

b 0 = low/medium (ISCED 1–4, 95, 97), high (ISCED 5, 6);

c 0 = no, 1 = yes.

d CASP-12 scale, higher scores indicate higher quality of life;

e EURO-D scale, higher scores indicate higher depression;

f Three-Item Loneliness Scale, higher scores indicate higher loneliness;

g higher scores indicate better health;

h ADL index, higher scores indicate more difficulties with ADL;

i Limitations in mobility, arm function, and fine motor skills, 0 = no, 1 = yes;

j Internet use within the last 7 days, 0 = no, 1 = yes;

k higher scores indicate better self-rated computer skills.

## 4. Discussion

The present study explored associations between individual characteristics and the implementation of special features or modifications in the homes of older adults in Europe. Logistic regression analyses revealed that health variables and functional abilities, such as limitations in ADL, mobility, and low grip strength, were important predictors for having respective modifications at home, which was also the case for social resources, such as having a larger social network and receiving care from a child outside the household.

A higher age was predictive of having modifications at home, which was also found in previous studies (12, 17, 18). For gender, education, and income, the body of research is more inconsistent [e.g., (10, 15, 19, 20)]. Gender effects were not found in our study, which was also reported in the recent release of the American Housing Survey (50). This can also be due to a confounding relationship with other study variables that is difficult to disentangle. For example, women reported significantly lower education and income, and better social resources (i.e., network size), but lower functional abilities (e.g., activities of daily living, mobility impairments) in our sample, and these indicators were, in turn, predictive for home modification in the one or the other direction. A higher education *reduced* the likelihood of home modifications in our study, but only in the young-old sample. This direction of association was also reported by Ishigami et al. (21), but, e.g., not by Meucci et al. (10), and could be due to the fact that higher education is often associated with better physical functioning in old age (51), which relates to a lower chance of home modifications. For participants with higher income, we found a higher chance of home modifications, which corresponds to some evidence, e.g., representative samples of noninstitutionalized US adults aged 65+ years (50, 52), but not to other, equally large studies (10, 15).

The importance of social resources (i.e., network size, and care from a child outside the household) that was indicated in the present study is in accordance with the findings of Ang et al. (25), Meucci et al. (10), and Peek et al. (23), who all reported evidence that informal caregivers are initiators of household modifications in order to facilitate everyday activities and prevent accidents.

Mental health variables could only explain a rather small share of variance. Based on the results, it could only be partially confirmed that mental health issues increase the likelihood of using technical aids in the home. In contrast to some previous studies (27, 28), persons who reported depressive symptoms were more likely to use assistive technology. A possible explanation is that this association is mediated by functional limitations since depression is associated with poorer health, which in turn is associated with a higher likelihood of using assistive devices.

Consistent with other studies, our results suggest that assistive devices are applied in the environment to maintain control and agency in daily life despite increasing functional limitations, which in turn maintains a high quality of life and satisfaction (29, 53). Moreover, fear of falling was a significant predictor of installing home modifications. This finding suggests that home safety and avoidance of accidents are important to many older adults. As functional indicators were highly significant for assistive device implementation, this could be interpreted as the individual strategy to adapt to the functional limitations and barriers in the environment.

The exploratively included factors of internet usage and computer skills were rather weak predictors in our analyses. In fourth age, only self-rated skills were slightly associated with the implementation of home modifications, while in third age, higher usage and skills were predictive for home modifications. ICT technologies are predominantly used for information seeking (e.g., reading the news), communications with others, or entertainment. In contrast, modifications in the home (e.g., ramps) serve more pragmatic purposes. In addition, older adults in our sample rated their overall computer competence as averagely poor, suggesting limited variance. Nearly half of those 65 years and older said they had never used a computer (47%).

### 4.1. Limitations

Our study has limitations that need to be acknowledged: the cross-sectional results do not allow causal interpretation and longitudinal analyses are needed to investigate if changes in individual characteristics, i.e., decreases in grip strength or increases in fear of falling, provoke the decision to install home modifications. Moreover, as the variance explanation was not high in total, there might be other factors that are relevant to the decision to have special features installed. For example, the length of residence in the respective household might increase the likelihood of having modifications implemented. The skewed distribution only allowed for a dichotomized and logistic approach, more detailed research is needed as well as explorative research among non-users regarding respective features and potential barriers toward adoption. As SHARE data only depict the existence of home modifications, but not actual usage, more fine-tuned assessments would provide further insights, i.e., on the frequency of use. Strengths of the study include the large and representative sample, the combined consideration of a variety of constructs clustered in different thematic blocks, and the comparison of two theoretically derived age groups. In addition, results of previous studies, mainly from the US or Canada, can be confirmed and transferred to older adults in Europe.

### 4.2. Conclusion

Data from Eurostat (2023) shows that the majority of people aged 65+ years live in their own households (with other persons or alone) (54). Home modifications bear the potential to enable aging in place for older people. It was the aim of our study (1) to analyze to what extent established technical aids and home modifications (automatically opening doors, ramps, grab bars, age-appropriate adaptations in bathrooms or kitchens, stair lifts, and alarm devices) are actually available in the households of older Europeans and (2) to explore which individual characteristics are substantially associated with the implementation of modifications in the household. We conclude that, although causal explanations are not possible, some findings relate to theoretical assumptions such as the environmental press theory. The installation of home modifications can be interpreted as a compensatory strategy that addresses gaps in the person-environment fit. In the seminal study of Lawton and Nahemow (33), as well as in later work in the field of environmental gerontology (35), it is argued that successful functioning is the result of a balance between the level of challenge occurring in the close environment and an individual's abilities to meet those challenges. The decision of older adults to have home modifications installed reflects an effort to regain this balance and is embedded in their personal, social, and physical context.

We perceive our study as a contribution to exploring the implementation of technology-based home modifications in Europe. Future research could be designed to uncover the entire implementation process and expand the effects of various technical aids on health. Thus, future studies could be able to provide more scientific knowledge for older adults, but also informal caregivers, on which aids are particularly effective. In addition, future studies should examine previously researched mediators and moderators from health psychology that might impact the implementation of assistive devices. One potential mediator between functional limitations and assistive technology devices in the home is knowledge and information about what modifications are available and what steps are required to obtain reimbursement. Another potential mediator variable is accessibility to technical aids and modifications. There might be a lack of options to acquire certain assistive devices in rural settings amplified by a lack of internet access at home. Therefore, future studies regarding aging in place should examine the extent to which place of residence is related to assistive technology implementation.

Our analyses may also provide some indications for interventions in public health, although we emphasize that home environments are a context of complex and multilayered interactions (35). The positive associations of social resources and functional limitations for implementing assistive devices suggest that facilitating and hindering factors of home modifications differ among older adults. As social resources were also quite strongly related to the likelihood of implementing technical aids, older persons with few social contacts seem more vulnerable in terms of aging in the community. For socially isolated individuals, strengthening the social network in the community can be helpful to encourage social interaction with other persons who already experience modifications. Other older adults with functional limitations, e.g., in personal care, will benefit from assistance in selecting an adequate assistive. At the same time, public health stakeholders are responsible to provide better and low-threshold information about the possibilities of household modifications and to make them financially accessible. Awareness of these entangled factors is needed in order to provide tailored support that may facilitate aging in place through the use of technical aids. Therefore, the article underlines the importance of public health responses concerning the heterogeneity of older households.

## Data availability statement

Publicly available datasets were analyzed in this study. This data can be found here: http://www.share-project.org/data-access.html?L=.

## Author contributions

LS was responsible for conceptualizing the outline, statistical models, data analysis, and discussion. MW participated in each part of the manuscript development and contributed to data analysis and the discussion. HB organized data analysis, contributed to the discussion, and facilitated the creation of the manuscript. AF prepared the introduction, the theoretical part, and the policy background and contributed to the discussion. All authors shared the preparation of the conclusion and made comments, suggestions, and corrections to the rest of the article. All authors contributed to the article and approved the submitted version.
